# Role of Imaging in Diagnosis and Management of COVID-19: A Multiorgan Multimodality Imaging Review

**DOI:** 10.3389/fmed.2021.765975

**Published:** 2021-11-08

**Authors:** Vinithra Varadarajan, Mahsima Shabani, Bharath Ambale Venkatesh, Joao A. C. Lima

**Affiliations:** Department of Cardiology, Johns Hopkins School of Medicine, Baltimore, MD, United States

**Keywords:** biomarkers, imaging, management, diagnosis, COVID-19

## Abstract

In this pandemic of Coronavirus disease 2019 (COVID-19), a vast proportion of healthcare resources, including imaging tools, have been dedicated to the management of affected patients; yet, the frequent reports of unknown presentations and complications of disease over time have been changing the usual standard of care and resource allocation in health centers. As of now, we have witnessed multisystemic symptoms requiring the collaboration of different clinical teams in COVID-19 patients' care. Compared to previous viral pandemics, imaging modalities are now playing an essential role in the diagnosis and management of patients. This widespread utility of imaging modalities calls for a deeper understanding of potential radiologic findings in this disease and identifying the most compatible imaging protocol with safety precautions. Although initially used for respiratory tract evaluation, imaging modalities have also been used for cardiovascular, neurologic, and gastrointestinal evaluation of patients with COVID-19. In this narrative review article, we provide multimodality and multisystemic review of imaging techniques and features that can aid in the diagnosis and management of COVID-19 patients.

## Introduction

Coronavirus disease 2019 (COVID-19), caused by a novel enveloped single-stranded RNA virus, SARS-CoV-2, has emerged as a pandemic leading to a global public health crisis of the unprecedented magnitude of mortality and morbidity (1, 2). The viral surface spike proteins bind to the human angiotensin-converting enzyme 2 (ACE2) receptor, expressed in the alveolar cells, vascular endothelium, and enterocytes of the intestine (3, 4). According to WHO guidelines, COVID-19 patients were divided into mild, moderate, severe, and critical stages based on symptoms, clinical signs, laboratory results, and imaging (5). The clinical spectrum of COVID-19 ranges from asymptomatic carriers who can transmit the virus to mild clinical upper respiratory infection, which can progress to an acute respiratory distress syndrome (ARDS) in critically ill patients (6). The most common symptoms in hospitalized patients were fever, dry cough, shortness of breath, nausea/vomiting, and diarrhea. In addition, anosmia, dysgeusia, and gastrointestinal symptoms were also reported among hospitalized patients (7). About one-quarter of hospitalized patients require intensive care treatment, most commonly due to hypoxemic respiratory failure (8). More than half of the ICU patients require mechanical ventilation. The clinical characteristics and complications of COVID-19 are not limited to pulmonary symptoms but include myocardial, renal, liver, thromboembolic manifestations, and neurological events (7). It is believed that SARS-CoV-2 causes direct viral tissue damage to extrapulmonary organs by attaching to ACE2 receptors. In addition, endothelial damage, thrombo-inflammation, and immune response dysregulation also contributed to the extrapulmonary manifestations of the disease (9).

Early detection and management of the disease are challenging as clinical symptoms are non-specific, and disease progression is quite rapid (8). The first-line diagnostic test of COVID-19 is carried out by real-time reverse-transcriptase PCR (RT-PCR) test from a nasopharyngeal swab, which reportedly has high specificity but low sensitivity ranging from 80 to 90% due to insufficient viral load or failure of nucleic acid extraction (10, 11). Due to a high number of false-negative results and non-specific biomarkers, radiological imaging has become a crucial tool in the early detection and risk stratification of COVID-19 disease. Given the impact of systemic inflammation and procoagulant activity of the disease, multimodality imaging can also aid in phenotyping the organ injury and dysfunction due to COVID-19. Thus, comprehensive insight into the imaging hallmarks of COVID-19 is mandatory for early diagnosis, stratifying the disease severity, effective treatment, and identification of potential sequela of this infection. On the other hand, comprehensive multimodality imaging cannot be employed due to the strict safety precautions required in the management of these patients, such as patient transfer and infection control measures. Hence, this narrative review sets out to explore the role and potential of multimodality imaging techniques in COVID-19 and outline the key findings in each modality. We searched Embase, PubMed, Cochrane library, and Web of Science databases through July 2021. No language restrictions were applied. Keywords of “lung imaging in COVID-19,” “brain imaging in COVID-19,” cardiovascular imaging in COVID-19, “lung injury in COVID-19,” “RV dysfunction in COVID-19,” “abdominal imaging in COVID-19,” “acute and chronic effects of COVID-19,” “diagnosis and management of COVID-19” were utilized. We manually searched the reference lists of included articles and relevant reviews. We present the following article in accordance with a focus on diagnostic imaging implementation in North America.

## Role of Biomarkers to Inform Imaging Decision Making

Circulating biomarkers play a key role in the clinical judgment as it reflects organ injury, inflammation, dysfunction, and organ damage, which informs the management of the disease. Biomarkers have also been found to be the predictors of longer hospital stay, need for ICU admission, the onset of ARDS, and mortality (12, 13). In the acute stage of disease, markers of inflammation such as C-reactive protein (CRP), ferritin, lactate dehydrogenase, and coagulopathy such as prothrombin times, D-dimer were reported to be elevated in most patients in addition to thrombocytopenia and lymphopenia (7). Cardiac biomarkers of myocardial injury (troponin) and myocardial stress (NT-proBNP) have been reported in patients with myocardial stress/injury related to COVID-19 and are not specific to acute heart failure or myocardial infarction (12). Similarly, even though the elevation of liver function tests may be non-specific, it is commonly seen in other viral pneumonia and may indicate a liver injury and dysfunction (7). As the involvement of extrapulmonary organs is a major adverse prognostic finding in COVID-19, the links between circulating biomarkers and organ dysfunction make it tempting to regard them as a valuable tool for decision making regarding the need for imaging. It is appropriate to interpret that the need for imaging can be ignored if the biomarkers are mildly elevated and can be utilized in marked elevation as in severe COVID-19.

## Role of Imaging in Diagnosis

### Initial Diagnostic Approach

#### Chest Radiography

Even though chest radiography is of little diagnostic value in the early stages due to high false-negative results, it has remained a technique often used for screening and disease severity assessment modality (11, 14). Equipment portability with imaging feasibility in the patient's room has made radiography favorable. A portable anteroposterior radiograph provides adequate information on lung findings associated with COVID-19, in addition to the assessment of lines and tubes and potential complications, such as pneumothorax, and maybe the only possible modality in severe infections.

**Figure 1:**
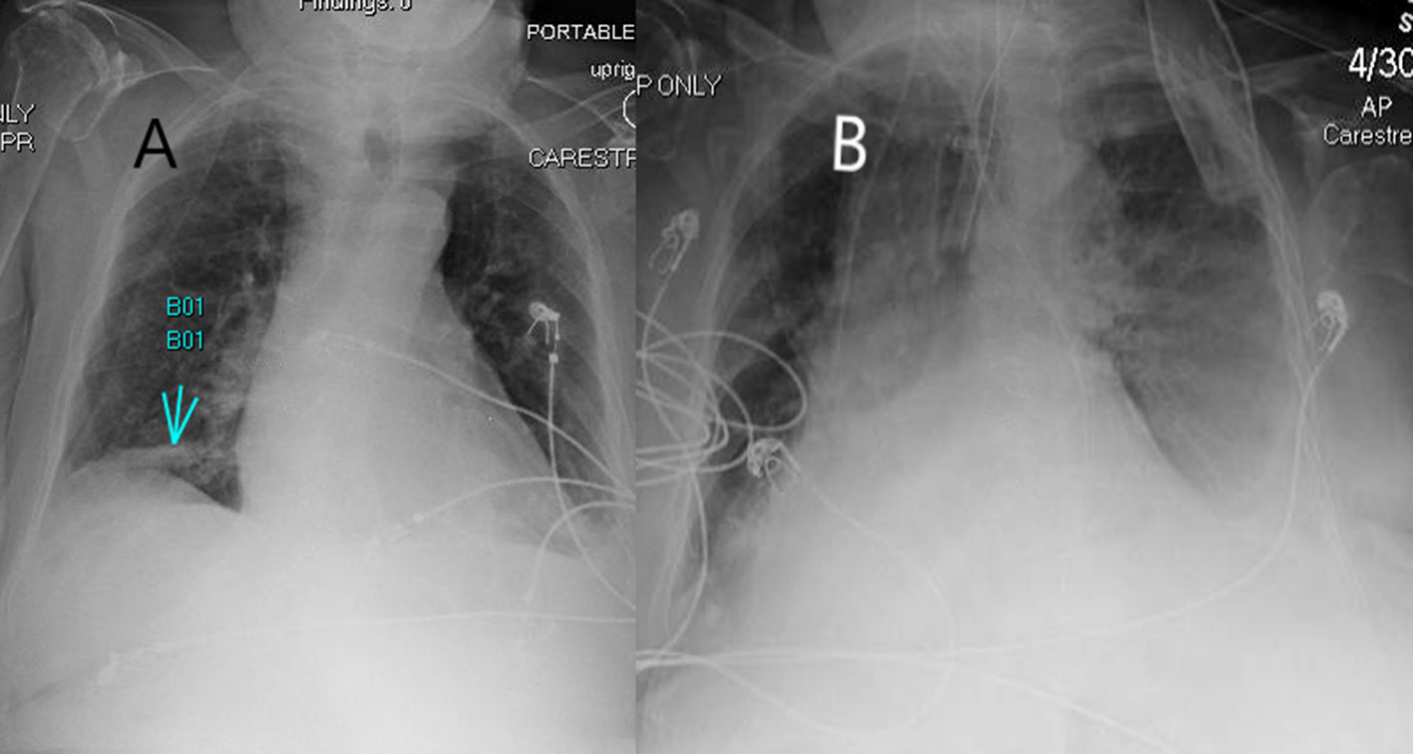
A 82-year-old woman presented to the emergency department with cough, shortness of breath, altered mental status. **(A)** X-ray showing a right lower lobe infiltrate (arrow) on day 1 and tested positive for COVID-19 using PCR. Later, due to worsening respiratory function and confusion, the patient was admitted to the intensive care unit and was intubated. **(B)** Image showing small left pleural effusion with worsening infiltrates.

At baseline, radiograph findings mostly include consolidation followed by hazy opacification (10, 11, 15). The opacification was mostly seen in peripheral regions close to the pleura and lower lung zones with unilateral or bilateral involvement of the lungs (11, 16). Reports showed that as the disease progressed, the opacities increased in size and density, with multilobar involvement gradually leading to diffuse opacity, consolidation, and thickened interlobular septa as seen in the setting of ARDS (Figure 1) (10). Chest radiograph findings in COVID-19 infection may overlap with other viral pneumonia and, as a result, have lower sensitivity (69%) than initial RT-PCR testing (11).

#### Chest Ultrasound

**Figure 2:**
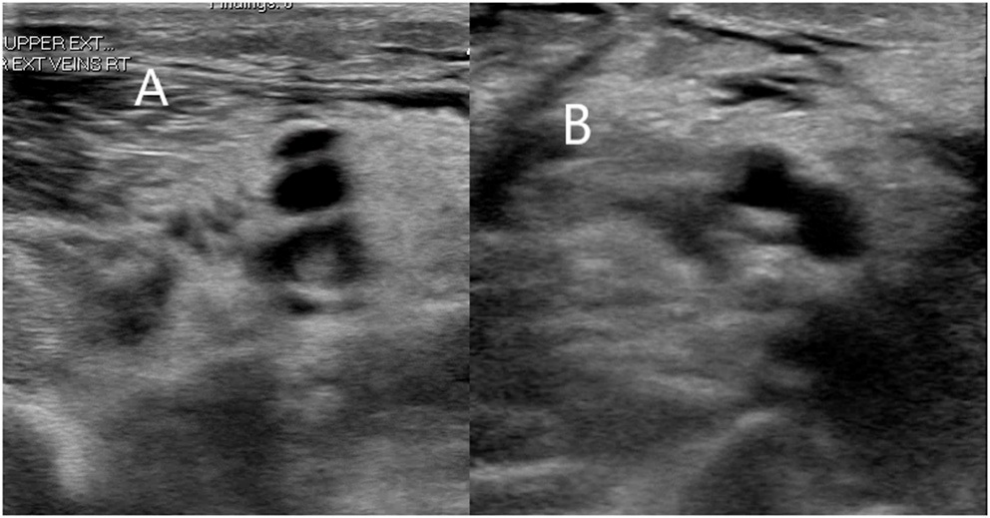
A 70-year-old man in the intensive care unit had right arm swelling after 1 week of diagnosis of COVID-19. Doppler ultrasound of the right arm showing occlusive thrombus within right brachial **(A)** and basilic veins **(B)**.

Lung ultrasound is easily accessible and widely used to diagnose, monitor, and manage patients in intensive care and emergency settings. Lung ultrasound has a higher sensitivity (94%) and specificity (85%) with a positive predictive value of 87% and a negative predictive value of 93% in diagnosing viral pneumonia, which leaves ultrasound as a potential assessment tool in COVID-19 infection (17, 18). Typical ultrasound findings in COVID-19 included pleural thickening, subpleural consolidation, B lines (multifocal or confluent), non-lobar and trans-lobar consolidation with air bronchograms, and small localized pleural effusions (17, 19). In the earlier symptomatic phase, pleural thickening with unilateral or bilateral B lines and spared areas were observed (19). In later stages, subpleural consolidations and visible air bronchograms were more dominant. In the recovery phase, there has been a regression of prior findings along with the re-emergence of A-lines (17, 19). Low cost, ease of use, higher accuracy compared to chest X-ray (CXR), no risk of radiation exposure, and repeatability made lung ultrasound widely adopted in hospitals. The American Society of Echocardiography (ASE) proposed an algorithm and list of potential findings for the utility of Point-Of-Care UltraSound (POCUS) in the cardiac, lung, and vascular examination of patients with suspected or confirmed COVID-19 (Figure 2). The real-time feature, flexibility, and multiorgan application of POCUS have made it a proper initial diagnostic modality and an assessment tool for the hemodynamic status of critically ill patients (20). Furthermore, wireless ultrasound devices are now substituting physician stethoscopes for lung auscultation, regarding probe-mediated patient contact and ease of disinfection (21).

#### Computed Tomography

**Figure 3:**
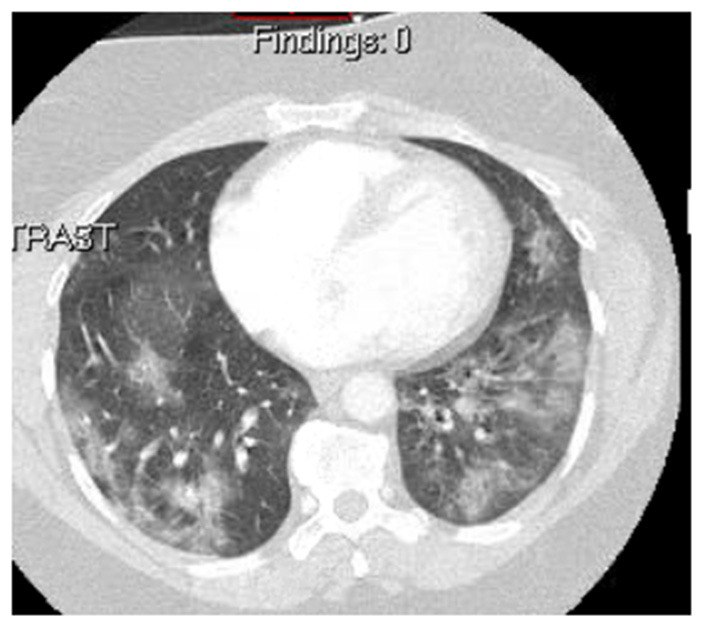
A chest CT of a 35-year-old woman with no significant past medical history presenting with progressive dyspnea, chest pain, and hemoptysis showing typical ground-glass opacities and consolidation, which is seen in COVID-19. The patient was confirmed of COVID-19 with PCR testing.

Although chest radiograph resembles CT findings, CT is more sensitive in detecting the abnormalities with a sensitivity of 98% (11, 22). Typical chest CT findings in patients with COVID-19 include ground-glass opacity (GGO) (98%), consolidation (64%), reticular and crazy-paving pattern (36%), predominantly in a bilateral, peripheral, posterior, and multilobar pattern (10, 11, 23–25). In earlier stages, GGO, hazy regions with increased density, and absence of broncho-vascular margins with a subpleural involvement of lower lobes either unilaterally or bilaterally have been reported to be the main CT findings (26, 27). Multifocal, patchy, or segmental consolidations have been considered as indicators of disease progression to the severe stage (25, 27, 28). In the peak of the disease, GGOs with a crazy-paving pattern, a thickened interlobular septa, and lines resulting from alveolar edema and interstitial inflammatory of lung injury were observed (10, 15, 27, 28). During the course of the disease and with aggravating symptoms, denser and diffusely distributed consolidation with more reticular configuration and thickened pulmonary interstitium due to lymphocyte infiltration were hallmarks (10, 26, 28). There were resolutions of consolidations and crazy-paving patterns in the healing stage with extensive GGO (Figure 3) (26, 27). Except for a peripheral distribution and involvement of upper and middle lobes in COVID-19, imaging findings overlap with other non-COVID 19 pneumonia and are not specific (29). Currently, the American College of Radiology (ACR) does not recommend CT as a first-line diagnostic test for COVID-19 patients and limits CT utility to symptomatic and hospitalized patients with specific clinical indication, conditional to appropriate infection control procedures. However, decision making based on chest CTs is still an option only in facilities with a lack of access to sufficient testing (30).

### Advanced Imaging Techniques

#### Magnetic Resonance Imaging

Due to strict infection control measures, pulmonary MRI of COVID-19 is quite challenging and has not been well established except for a few case reports. MRI performed for non-pulmonary indications demonstrated incidental pulmonary findings consistent with CT (10). Pulmonary parenchymal focal zones can be visualized with abnormal intensity signals using T1 and T2 sequence as “cloudy sky sign” in acute settings, which can be correlated with GGO in CT (31).

#### Fluorine-18-Fluorodeoxyglucose Positron Emission Tomography/CT (^18^F-FDG-PET/CT)

Another imaging modality, ^18^F-FDG PET/CT is a non-invasive imaging method that identifies sites of inflammation and infection based on their higher metabolic demands (10). Few case studies reported increased uptake of ^18^F-FDG with a standardized uptake value of 4.6–12.2 in patients with peripheral GGO and lung consolidations detected by chest CTs (32, 33). Notably, ^18^F-FDG PET/CT revealed an increased nodal uptake despite the absence of lymphadenopathy in COVID-19 (32, 33). Although ^18^F-FDG PET/CT cannot be used in emergency settings, it can play a complementary diagnostic role in COVID-19, especially when the symptoms are non-specific and diagnosis is challenging. Table 1 summarizes the pulmonary features of COVID-19.

**Table 1:** Pulmonary imaging features of COVID-19.

**Modality**	**Findings**
Radiograph	**Acute:** Basal consolidation with hazy opacification; unilateral/bilateral. **Progression:** Diffuse and irregular opacification as seen in ARDS.
CT	**Acute:** Ground-glass opacities (GGOs) unilateral or bilateral. **Progression:** Multifocal, patchy, or segmental consolidation. GGO with crazy-paving pattern. **Severe:** Reticular configuration, diffuse consolidation. **Recovery:** Progressive resolution. Disappearance of signs.
USG	**Acute:** Pleural thickening, subpleural consolidation, multifocal B lines. **Progression:** Subpleural consolidation with visible air bronchograms. **Recovery**: Re-emergence of A-lines.
MRI	**Acute:** Cloudy sky sign with abnormal intensity using T1 and T2 sequences corresponding to GGO on CT.
^18^F-FDG PET/CT	**Acute:** Increased uptake corresponding to GGO on CT. Increased nodal uptake.

## Role of Imaging in Management

Even though a typical presentation of COVID-19 was pulmonary symptoms, clinicians were frequently consulted to rule out cardiovascular, cerebrovascular, thrombogenic, and abdominal manifestations. Based on history, physical examination, biomarkers, ECG, and imaging modalities, clinicians can make an informed decision on the management.

## Cardiovascular Imaging

The cardiovascular complications of COVID-19, including myocardial infarction, myocarditis, acute-onset heart failure, thromboembolism, and cardiac arrest, are of great concern due to the higher case fatality rate observed in this subgroup of patients (34, 35). Myocardial involvement was associated with poor prognosis and more complicated adverse events, and therefore, there is a need for guidance on significant cardiac findings associated with COVID-19 (35–38). Table 2 summarizes the cardiovascular imaging findings of COVID-19.

**Table 2:** Cardiovascular imaging features of COVID-19.

**Modality**	**Acute findings**	**Progressive findings**
Echocardiography	Increase in ejection function.	RV dilation with or without dysfunction followed by LV dysfunction. Worsening RV longitudinal strain. LV apical ballooning with hyperkinetic basal segments.
CT	Reveals a filling defect due to pulmonary embolus, thrombus in the aorta. Can be used to assess plaque destabilization and progression.	
MRI	Increased wall thickness, severe LV dysfunction, diffuse biventricular hypokinesis of apical segments.	Diffuse interstitial myocardial edema by increased native T1 and ECV. LGE showed a diffuse pattern suggestive of acute myocarditis.

### Initial Diagnostic Approach

#### Echocardiogram

In most COVID-19 patients, there have been signs of a hyperdynamic cardiac function, assumed to be due to systemic inflammation (39, 40). There have also been several reports of stress-induced (Takotsubo) cardiomyopathy observed as left ventricle (LV) apical ballooning with hyperkinetic basal segments (39, 41, 42). Other frequently observed echocardiographic signs were right ventricle (RV) dilation (the D-sign) with or without dysfunction, which is a poor prognostic factor and not just a sign of pulmonary embolism (PE), followed by LV diastolic and systolic dysfunction, pericardial effusion, and mild valvular heart disease (43–45). The majority of severe COVID-19 patients were found to have RV dilation/dysfunction along with elevated D-dimer, CRP, troponins, and cytokines (46). RV longitudinal strain was also found to provide prognostic information on COVID-19 (46, 47). In critically ill patients, hypoxic respiratory failure-induced pulmonary circulation injury causes severe RV dysfunction, which has a poorer prognosis and increased mortality in COVID-19 (46, 48). Consistently, severe global LV dysfunction and wall motion abnormalities have been observed in complicated cases of COVID-19 (36, 39).

Given the hypercoagulability induced by this virus, a phenomenon of pulmonary thrombosis *in situ* due to pulmonary involvement and local inflammation of the lung, rather than the migration of peripheral embolus, has been noted, which may suggest a different pathophysiologic mechanism of PE in this disease compared to PE described in other circumstances (49–51). Although echocardiography is not a sensitive tool in the early detection of pulmonary embolism, the secondary RV dysfunction signs, including severe free wall hypokinesia or akinesia with apical sparing (McConnell sign), shorter pulmonary acceleration time, and paradoxical interventricular septal movement, were prognostic markers for PE (10, 52, 53).

Echocardiography is a safe, non-invasive technique that allows evaluation and quantification of global and regional cardiac function. The ASE guideline has recommended that POCUS, FoCUS (Focused Cardiac Ultrasound Study), UAPE (ultrasound-assisted physical examination), and bedside critical care echocardiography are preferred diagnostic modalities for CV manifestations of COVID patients (54).

### Advanced Imaging Techniques

#### Magnetic Resonance Imaging

Even though cardiac magnetic resonance imaging (CMR) is a safe, non-invasive, and preferred imaging modality in the quantification and characterization of myocardial tissue, the risk of contamination and imaging feasibility makes it challenging in acute settings. Increased wall thickness, diffuse biventricular hypokinesis of apical segments, severe LV dysfunction, and circumferential pericardial effusion was seen in the acute phases of infection (36). Moreover, the increased signal intensity of LV myocardium in T2-weighted images (T2WI) or native T1 and the increased extracellular volume (ECV) was suggestive of interstitial myocardial edema to identify the presence of myocardial inflammation in these patients (55). Late gadolinium enhancement (LGE) imaging showed diffuse-pattern involvement with predominantly subepicardial distribution, extended to the entire biventricular wall, fulfilling all Lake Louise criteria for the diagnosis of acute myocarditis (36, 55, 56). Dedicated protocols with rapid acquisition to assess ventricular morphology and function (cine-images) and to detect myocardial edema (T2WI) in acute settings are encouraged (57). On the other hand, decreased RV ejection fraction, cardiac output, stroke volume suggest RV myocardial edema, fibrosis, and impaired contractile function in patients recovered from COVID-19 (46).

#### Computed Tomography

Computed tomography angiography (CTA) is a rapid, non-invasive, broadly accessible, and highly accurate imaging method that can be utilized in hospitalized patients with elevated cardiac biomarkers. CTA has been replacing coronary angiography in several clinical settings and is an effective goalkeeper for invasive procedures, lowering costs, radiation, and adverse effects without compromising the patient outcome. CTA has also aided in visualizing the filling defects due to pulmonary embolus, or thrombus in the aorta, and has been preferred over (16, 58, 59). Additionally, in accordance with guidelines, CT pulmonary angiography should be performed to rule out pulmonary embolism if supplementary oxygen was needed in COVID-19 patients with limited disease extension or when unenhanced CT findings could not explain the severity of the respiratory failure (60). Late contrast-enhanced cardiac CT has also been recommended as an alternative modality using dual-energy CT for myocarditis diagnostic when CMR use is not feasible, especially in patients with a planned concurrent lung CT (61). Findings in favor of myocarditis in cardiac CT are myocardial edema defined by a subepicardial late iodine enhancement, and a sub-endocardial perfusion defect, as observed in COVID-19 cases (62).

## Cerebrovascular Imaging

The neurological manifestation of COVID-19 has been pleiotropic ranging from dizziness, anosmia, and dysgeusia in initial stages to immune-mediated vasculitis, stroke, and cranial neuropathies, and in full-blown disease, encephalomyelitis (63). In case of neurological emergencies like an acute stroke in COVID-19 patients, individual CT devices capable of angiography (CTA) and perfusion, multimodal MRI, and endovascular intervention units would be required to provide proper care in a rapid time while not interfering with non-COVID patients with the same acute presentations. Given this need, multiple stroke management algorithms have been designed and tested in multiple centers (64–66). Table 3 summarizes the imaging findings of the brain in COVID-19.

**Table 3:** Neurovascular imaging features of COVID-19.

**Modality**	**Acute findings**	**Progressive findings**
MRI	FLAIR images with hyperintensity of the cortex and bilateral olfactory bulbs with normal volume.	Infarct of cerebral arteries with MRA revealing cerebral artery stenosis. Diffuse hyperintensities of bilateral temporal lobe and thalamus. Rim enhancement on T1 suggesting acute necrotizing encephalopathy.
CT	Extensive hemorrhage with subarachnoid and interventricular hypodensity. Acute/subacute infarcts can be seen.	Hypoattenuation localized to bilateral medial thalami. Cerebral vein thrombosis.

### Initial Diagnostic Approach

#### Computed Tomography

**Figure 4:**
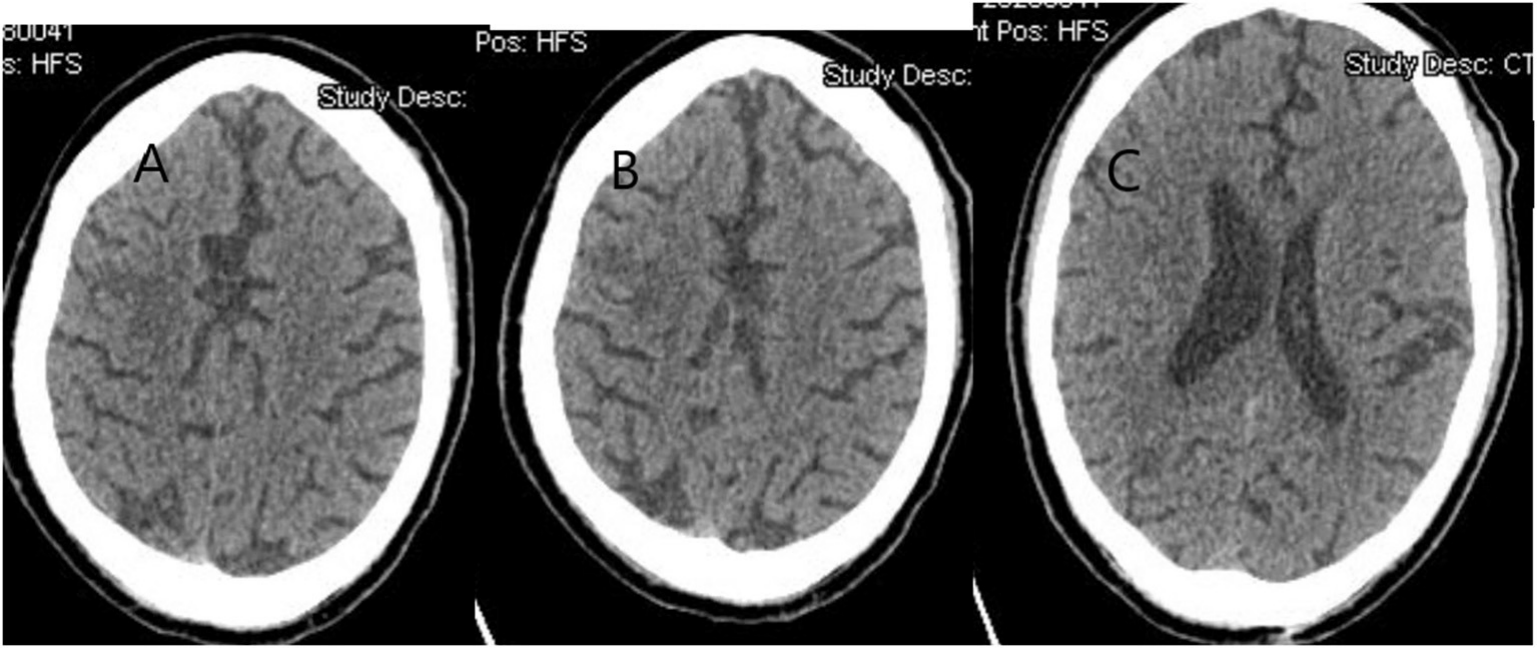
A 68-year-old man complained of acute weakness of the left side of her body 3 days after being diagnosed with COVID-19. Brain CT without contrast shows multiple foci of infarcts in the right middle cerebral artery involving the right frontal, parietal, and temporo-occipital junction.

In the acute stages of COVID-19, brain CT showed no abnormalities, but later in the course of the disease, extensive acute subarachnoid and intraventricular hemorrhages along with cerebral swelling and hypodensity were reported in some patients (63, 67). In other studies, localized hypoattenuation in bilateral medial thalami causing acute necrotizing encephalopathy and cerebral vein thrombosis was visualized in brain CTs (63, 67). In patients with preexisting cardiovascular risk factors, there is a higher prevalence of acute ischemic strokes (Figure 4) (68).

In some instances, COVID-19 patients presented with varying degrees of peripheral neuropathy, especially Guillain–Barre syndrome (GBS). Neuroimaging has been recommended to be deferred in the case of neuromuscular disorders unless COVID-specific precautions were not required (69). In GBS, spinal and brain MRI showed the enhancement of caudal nerve root and bilateral facial nerve, respectively (63, 70).

#### Magnetic Resonance Imaging

In the earlier stages of the disease in a patient with anosmia, brain MRI fluid-attenuated inversion recovery (FLAIR) showed cortical and bilateral olfactory bulb hyperintensity with normal sizes of olfactory bulbs (63, 71). As the disease progressed, the inflammatory and hypercoagulable state resulted in clinical strokes with brain MRI showing acute infarct of cerebral arteries and magnetic resonance angiogram (MRA) revealing stenosis of cerebral arteries (63, 67, 72). In severe stages of the disease, brain MRI findings included diffuse hyperintensities of bilateral temporal lobe and thalamus, as well as non-confluent multifocal white matter hyperintense lesions on FLAIR and extensive isolated white matter microhemorrhages. There have also been reports of internal hemorrhages indicated by hypointense signals on susceptibility-weighted imaging and rim enhancement on T1-weighted post-contrast imaging suggestive of acute necrotizing encephalopathy (63, 67, 73–75).

## Abdominopelvic Imaging

Although a minority of patients have presented with digestive-only manifestations, almost half of the confirmed cases of COVID-19 had both respiratory and digestive symptoms, with increased severity of the disease in those with gastrointestinal-specific manifestations, which mandates further evaluation by imaging (76). Table 4 summarizes the abdominopelvic imaging features of COVID-19.

**Table 4:** Abdominopelvic imaging features of COVID-19.

**Modality**	**GI**	**Pancreas**	**Liver**	**Renal**
Radiograph	Portal venous gas suggesting bowel infarction and perforation.			
USG		Voluminous pancreas without gall stones.	Gall bladder distension, GB wall thickening with GB sludge—acute cholestasis. Increased echogenicity of hepatic parenchyma—fatty liver.	
CT	**Acute:** Fluid-filled colon. **Progression:** Bowel wall thickening, pericolic fat stranding, pneumatosis intestinalis, mural hyperenhancement. Dense opacification of the intestinal wall.	**Acute:** Pancreatic edema, dilation of the pancreatic duct with focal necrosis. **Progression:** Duodenal/periduodenal inflammation.	Heterogeneous liver with GB thickening.	Edema of the renal parenchyma. Wedgeshaped low attenuation suggesting infarct.
MRI			Increased liver fat>10% in severe disease.	

### Initial Diagnostic Approach

#### Abdominopelvic Radiography

There were reports of portal venous gas suggestive of bowel infarction and signs of perforation in some cases (77).

#### Ultrasound

**Figure 5:**
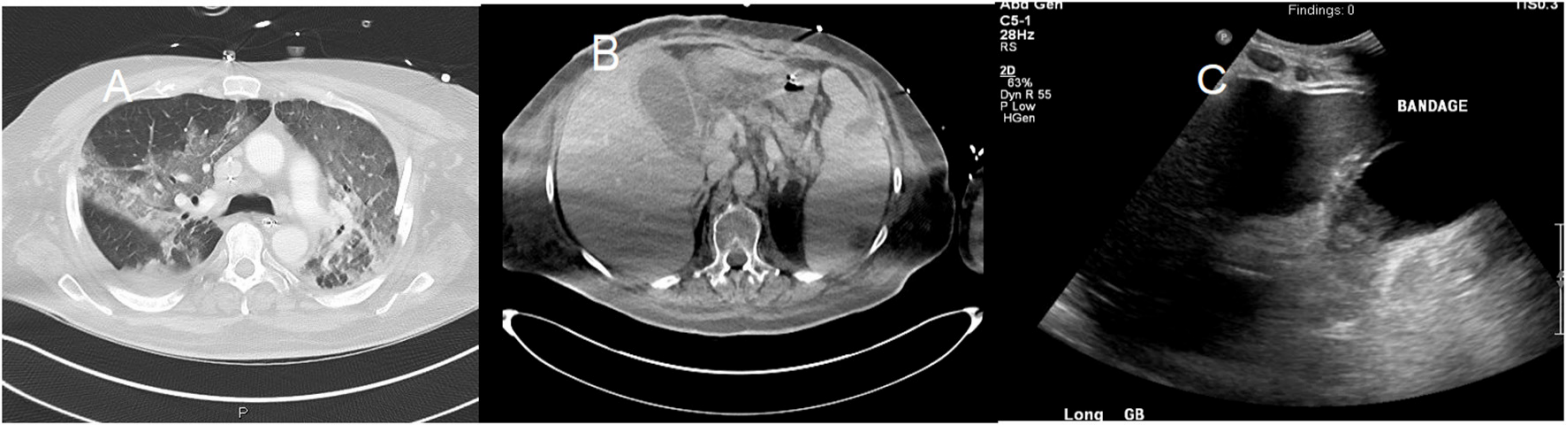
A 58-year-old man with a past medical history of obesity, post-primary coronary intervention, and coronary artery disease, got admitted to the intensive care unit with severe hypoxia and tachycardia. The patient tested positive for COVID-19. **(A)** Chest CT showed patchy airspace opacities with predominant ground-glass opacities seen in the upper lobes and consolidative opacities with air bronchograms in the lower lobes. **(B)** Abdominal CT showed hepatosplenomegaly with splenic infarcts and perisplenic fluid collections. Gallbladder wall thickening with trace pericholecystic fluid was also noted, suggesting acalculous cholecystitis. **(C)** An abdominal ultrasound image of the same patient showed moderately distended and thickened walls of the gall bladder.

Additionally, ultrasound findings also revealed gallbladder distension, wall thickening, and sludge reflecting acute cholestasis (Figure 5), as well as increased echogenicity of hepatic parenchyma obscuring periportal echogenicity as markers of fatty liver, and mild pancreatitis revealed as the voluminous pancreas (77–79).

### Advanced Imaging Techniques

#### Computed Tomography

Computed tomography-derived signs in patients with COVID-19 ranged from the fluid-filled colon in the early stages of diseases to more severe cases of local enterocolitis or even pancolitis with bowel wall thickening, small bowel distension, segmental bowel ischemia, pericolic fat stranding, pneumatosis intestinalis, mural hyperenhancement, and mesenteric hypervascularity (77, 80, 81). Due to parenchymal inflammation, density around the intestinal wall mimicking a GGO specific to COVID-19 can be noted in CT (81). Abdominal CT has also been a remarkable diagnostic tool for COVID-19-associated cholecystitis, hepatitis, and splenic infarcts (Figure 5). Typical CT findings in patients with pancreatic complications varied from mild pancreatic edema and dilation of pancreatic duct to focal necrosis of pancreas with distinct inflammation involving second and third parts of the duodenum and periduodenal space, which was suggested to be specific to severe stages of COVID-19 (78, 79, 82). Moreover, there have been reports of renal involvements in abdominopelvic CT scans of COVID-19 patients, including parenchymal edema and more specifically wedge-shaped low attenuation region in the superior pole of the kidney, suggestive of renal infarct due to thromboembolism (16, 37).

#### Magnetic Resonance Imaging

Liver injury in COVID-19 patients is believed to be a consequence of either mechanical ventilation and/or severe inflammation and stress-induced injury (83). One potential method for the quantification of hepatitis severity is hepatic T1 mapping MRI, in which liver fat proportions of more than 10% have been shown to be associated with the severity of COVID-19 infection (84).

## Discussion

This multiorgan multimodality narrative review provided comprehensive information on the burgeoning evidence of varied clinical phenotypes of COVID-19. Studies have reported incidental COVID-19 findings in the lung bases observed in the non-pulmonary imaging of patients with non-COVID indications, including acute abdomen or preoperative scans (85, 86). This highlights how non-pulmonary imaging could be as important as pulmonary imaging in the diagnosis and management of patients, given the unraveled nature of COVID-19. One of the major recommendations of ACR during this pandemic was the necessity for physicians to be able to recognize possible COVID-19-associated imaging findings even in patients with imaging not acquired for COVID-related complaints (30).

As COVID-19 is a new disease, research relies on retrospective data collected from patients with both imaging and RT-PCR done and hence subjected to selection bias. When translating these reported findings to clinical practice, clinicians should exercise caution and pay attention to the characteristics of each study population, as population differences can result in conflicting results in diagnostic accuracy of the modalities (87). Additionally, the challenges of COVID-19 have also caused alterations and significant changes to the workflow of clinicians and radiologists as the health and safety of healthcare workers come into play along with patients. Diagnostic imaging services have been time-consuming and complicated by the need for strict infection control and prevention practices developed to contain the risk of transmission and protect healthcare personnel (88). Hence, the decision to image suspected patients or COVID-19-positive patients is based on their impact on the improvement of patient status. The ACR recommends not to use any advanced imaging as a screening test and only to use radiography and ultrasound as a first-line tool (11, 30). Even though radiographs are insensitive for the detection of specific signs of disease, equipment portability can favor this imaging modality in triaging and monitoring disease (10, 88). Ultrasound and echocardiography can provide useful insights that can be correlated with CT findings but need to be employed with certain caveats to ensure the safety of the healthcare provider. Especially in critically ill patients, the use of POCUS by the clinical provider for disease management can be helpful as formal echocardiograms can increase exposure to care providers and can be challenging to coordinate.

There are a few limitations to our review paper. Since this is a narrative review, we might have introduced selection bias in selecting the articles and literature in this review. Second, as the aim of this review was not to critically appraise literature, we did not assess the quality and validity of the literature and the evidence based on the predefined protocol. Third, we could not address the diagnostic value and specificity of imaging findings as, to our knowledge, only a few clinical studies were published comparing COVID and non-COVID imaging findings. Further comparative analysis is required. Lastly, at the time of the review was written, not much was established about the long-term effects of COVID-19, and hence, not much could be described as the role of imaging in long-term COVID-19.

Given that most clinical presentations of COVID-19 have been non-specific and commonly observed in many other systemic diseases, this thorough review of reported imaging findings in this disease could be a potential guide for clinical practitioners, radiologists, and intensivists to diagnose early and improve the management of patients referred with these alarm signs. Since cycles of acceleration, suppression, and re-emergence of COVID-19 have been predicted, multimodality imaging can play a crucial role in diagnosing and stratifying patients in the event of a second wave. Future research is needed to establish the long-term sequelae of COVID-19 infection on multiorgan function.

## Conclusion

Imaging in complement with clinical examination and biomarkers can provide a detailed assessment and management of organ damage and dysfunction in COVID-19. In the era of COVID, all physicians should endeavor to carefully look for imaging features reported in COVID-19 patients, even in asymptomatic patients and in imaging other than chest radiographs, as patients with multisystem presentations have had unfavorable outcomes such as hospitalization, mechanical ventilation, and mortality.

## Author Contributions

VV: conceptualization, acquisition, and writing original draft. MS, BA, and JL: writing, reviewing, and editing. All authors contributed to the article and approved the submitted version.

## Funding

The writing group has been funded by the National Heart and Lung Blood Institute (NHLBI), part of the National Institute of Health.

## Conflict of Interest

The authors declare that the research was conducted in the absence of any commercial or financial relationships that could be construed as a potential conflict of interest.

## Publisher's Note

All claims expressed in this article are solely those of the authors and do not necessarily represent those of their affiliated organizations, or those of the publisher, the editors and the reviewers. Any product that may be evaluated in this article, or claim that may be made by its manufacturer, is not guaranteed or endorsed by the publisher.
